# Supplementary material for: A comprehensive wireless neurological and cardiopulmonary monitoring platform for pediatrics
Source: PLOS Digit Health. 2023 Jul 6;2(7):e0000291. doi: 10.1371/journal.pdig.0000291 (PMC10325120; doi:10.1371/journal.pdig.0000291)
Supplement: Attachment — Submitted filename: Response Xu PLOS Digital Health Final.docx [file pdig.0000291.s002.docx]

**Responses to Initial Reviewers from the Editors and Reviewers**

We would like to sincerely thank you for having taken time to review our submission and provide your valuable feedback. We believe our responses here reflect a substantial revision of the manuscript making it suitable for publication in *PLOS Digital Health*.

**Editor Comments**

1. Please send a completed 'Competing Interests' statement, including any COIs declared by your co-authors. If you have no competing interests to declare, please state "The authors have declared that no competing interests exist". Otherwise please declare all competing interests beginning with the statement "I have read the journal's policy and the authors of this manuscript have the following competing interests:"

**Author response**: We agree with this point.

**Manuscript modifications:** We provided a Competing Interests statement in our resubmission per the guidelines that were given.

2. Please amend your detailed Financial Disclosure statement. This is published with the article. It must therefore be completed in full sentences and contain the exact wording you wish to be published.

a. State the initials, alongside each funding source, of each author to receive each grant.
b. State what role the funders took in the study. If the funders had no role in your study, please state: “The funders had no role in study design, data collection and analysis, decision to publish, or preparation of the manuscript.”
c. If any authors received a salary from any of your funders, please state which authors and which funders.

If you did not receive any funding for this study, please simply state: “The authors received no specific funding for this work.”

**Author response**: We agree with this point.

**Manuscript modifications:** We amended the Financial Disclosure statement in our resubmission per the guidelines that were given. We stated that the funders had no role in study design, data collection and analysis, decision to publish, or preparation of the manuscript.

3. We do not publish any copyright or trademark symbols that usually accompany proprietary names, eg  ©, ®, ™  (e.g. next to drug or reagent names). Please remove all instances of trademark/copyright symbols throughout the text, including ® on pages 2, 3, 6, 7, 8, 10, 12, 13, 14, 22.

**Author response**: We agree with this point.

**Manuscript modifications:** We removed all instances of trademark and copyright symbols including in the pages mentioned.

The manuscript presents a commercial device assessment. As such, the novelty is limited by the impossibility to disclose the technical details of such a device. The authors should improve this, along with the statistical analysis presented in the manuscript. Is there a chance to enlarge the sample size? Please carefully address in a rebuttal letter and possibly in the manuscript the answer to the Reviewers criticisms.

**Author response:** Thank you for these comments and for your detailed consideration of our manuscript. We made significant changes to address these critiques. Our primary aim in this manuscript is to introduce a novel wireless EEG monitoring system that complements an existing USA FDA-cleared multimodal wearable sensor platform with comprehensive, data-driven validation.

We strengthened three key portions of the manuscript in response to reviewer feedback. First, we greatly expanded upon the Results section to provide greater technical detail, focusing on novel aspects of the system design. We also provided other important metrics requested by reviewers such as projected system cost, battery life and reusability. Second, we tripled the sample size by adding a feasibility study performed with the device in two other sites, including one in the LMIC setting, encompassing an additional 61 patients. This attests to the scalability of the system as well as its adaptability for use in a variety of environments. Third, we added further quantitative data analysis in the validation study cohort by performing power spectrum analysis of the EEG data collected in studies in which sleep was captured. We demonstrated that the influence of epileptiform spikes on the power spectra is concordant with prior work.

**Manuscript modifications:**

1. We expanded content in the *Results* section, highlighting further technical details:

“Figure 1 displays key aspects of the system’s sensor design. A four-layer flexible printed circuit board is fabricated for the ANNE EEG sensor that consists of a stack-up of copper, polyimide, coverlay, and soldermask. The sensor consists of the following; a system-on-a-chip (SoC, ISP1807, Insight SIP); high-precision and low noise analog-front-ends (AFE) for a maximum of 16 channels; and a power management unit for wireless charging and supplying power to the on-board electronics with a rechargeable Li-polymer battery (230 mAh). The SoC controls the two AFE with on-board low-noise PGAs and sigma delta ADCs to collect clinical grade EEG data, where each channel samples at 1 kHz with 24-bit resolution and 0.28 µVrms input noise. The sensor board is enclosed with a ruggedized plastic housing made of Acrylonitrile butadiene styrene (ABS) with 16 channel connectors to standard EEG electrodes.”

2. We added the following text to the start of second paragraph of the Results section:

“Panel A of Figure 2 provides an exploded schematic view of the device to highlight the underlying technology. ANNE EEG has a battery life of 8 hours between charges and a projected cost of $1,000 USD to allow for cost-effective operation in LMIC environments. The device is fully reusable.”

3. We added Figure 1, a block diagram of the system’s design.
4. We modified the abstract to specify pilot study of 91 patients at three centers, including one in the LMIC setting, specifying that 30 were part of a validation study and 61 were part of a feasibility study. We added details of each center’s IRB in the Study design and data collection subsection of Materials and Methods.
5. We added Table 1, Characteristics of Study Participants to encompass all 91 patients, as well as added content in the *Study design and data collection* subsection of Materials and Methods consisting of the following:

“We demonstrate performance, usability and satisfaction of ANNE® EEG, in a multi-center feasibility study as well as a single center validation study. Table 1 shows demographic characteristics of all study participants, with ages ranging from birth to 18 years.

In the single center validation study, the aim was to demonstrate signal quality equivalence compared to current clinical EEG systems in a clinical cohort of 30 patients. In one patient, ANNE® chest and limb modules were also added as a proof-of-concept demonstration. Table 2 shows demographic characteristics of validation study participants. All the assessed participants had an epilepsy related diagnosis, ranging from genetic syndromes to structural brain abnormalities such as cerebral injury from hypoxic ischemic encephalopathy. Recruitment included patients ages 0-18 who presented to the Lurie Epilepsy Monitoring Unit (EMU) as part of a clinically indicated 4-hour EEG study. There were no other exclusion criteria. Patients were screened by a pediatric epileptologist (J.N.W.) through review of the electronic medical record.”

6. We expanded upon the *Discussion* section to comment the increased sample size as follows:

“We present an innovative comprehensive wireless neurodevelopment monitoring system that adds neuronal activity measurement via EEG to previously validated chest and limb modules. Pilot studies at an academic quaternary pediatric care center demonstrate the practicality and feasibility of ANNE EEG to conduct electroencephalography studies with high levels of accuracy, validated via both quantitative and qualitative metrics, compared to the gold standard system. Furthermore, we also demonstrate the feasibility and scalability of the system for use in neonates in the LMIC setting. Previous work has already illustrated the performance and safety of the ANNE Chest and ANNE Limb sensors for cardiopulmonary monitoring in the LMIC setting.”

7. We modified the following paragraph of the *Discussion* section to account for our study’s increased sample size and limitations with the additional statistical analysis:

“Our validation study has a few limitations that require further investigation prior to widespread deployment, particularly in medically underserved regions. While 30 subjects is adequate for validation of a novel EEG device validation [16] this is a relatively low number; we intend to increase this within our own institution. We also intend to expand upon the feasibility studies performed at both HIC and LMIC settings with future iterations of the system. Smaller form factors and electrode solutions that more specifically meet the needs of premature and ultra-low birth weight infants [36] would also be ideal for this vulnerable population.

While the power spectra trends displayed in Figure 7 were not statistically significant due to the small subgroup sample size, results are consistent with prior literature investigating spectral power in patients with epilepsy. We intend to investigate this trend further in future studies.”

8. We displayed results of the multi-center feasibility study in figure 4, adding the subsection to *Results* entitled “The EEG system captures key elements of neonatal sleep in HIC and LMIC settings”:

“Figure 4 displays typical graphoelements of neonatal sleep activity measured by ANNE EEG. Analysis of quiet and active stages of neonatal sleep has been shown to predict neurodevelopmental outcome [21]. Figure 4A displays features of quiet sleep in the HIC setting, and figure 4B displays separate features of active sleep in the LMIC setting.”

9. We added figure 7 and supplementary table 1. These display the results of power spectrum analysis and subsequent statistical analysis, respectively.

We expanded the *The EEG system demonstrates high levels of accuracy via qualitative and quantitative metrics in a pediatric cohort* subsection of the results section, consisting of the following paragraph:

“Further quantitative analysis (see: Methods for details of power spectrum analysis) was also performed for patients whose sleep activity was recorded via ANNE EEG. Figure 7 compares the distribution of EEG power (absolute broadband power and relative delta frequency) in sleep without epileptiform spikes versus sleep with epileptiform spikes. These findings are concordant with prior studies [25,26] that showed an increase in both these metrics for individuals with epilepsy.”

Respectively, we also added the following paragraph to the *Data Analysis* subsection of the **Materials and Methods** section:

“Relative power spectrum analysis was calculated for each channel in a subset of the validation study cohort. Sleep epochs were chosen to minimize the effects of movement artifact. The same representative 30 minute epochs of continuous sleep were used as in the alpha-delta ratio calculations. Power spectra for each of the 16 channels were divided into the following physiologically relevant frequencies; delta (0.5 to 4 Hz), theta (4 to 8 Hz), alpha (8 to 13 Hz), and beta (13 to 30 Hz). Each of these frequency bands were then averaged across all channels along with the total power for the epoch (Supplementary Table 1). Boxplots were then generated to compare relative delta ratio and total power between the subgroup with epileptiform discharges versus the subgroup without epileptiform discharges.”

**Reviewer 1:**

In this study, the authors tried to validate a novel wireless monitoring system by showing its ability to record high-quality cerebral electric activity. ANNE® EEG which can record 16 total recording channels was used in this study. Although the idea is novel analysis results are not convincing enough to accept it as a full journal paper. A sample size of 30 subjects with varying ages is not justified to me. I have some alternative suggestions for the authors to think about if they want to resubmit the paper.

1. Increase the sample size uniformly so that there is at least 3 subjects are included for each year of neurodevelopment.

**Author response**: We increased the sample size to a total of 91 subjects and stratified them by USA FDA-defined age ranges for medical devices; neonates (0 to 28 days), infants (> 28 days to 2 years), children (2 to 11 years), and adolescents (12 to 21 years). While we were unable to have a comparable number of participants across the defined epochs of neurodevelopment, we demonstrate that we included at least six participant in each one.

**Manuscript modifications:**

1. We added Table 1, Characteristics of Study Participants to encompass all 91 patients, as well as added content in the *Study design and data collection* subsection of Materials and Methods consisting of the following:

“We demonstrate performance, usability and satisfaction of ANNE® EEG, in a multi-center feasibility study as well as a single center validation study. Table 1 shows demographic characteristics of all study participants, with ages ranging from birth to 18 years.

In the single center validation study, the aim was to demonstrate signal quality equivalence compared to current clinical EEG systems in a clinical cohort of 30 patients. In one patient, ANNE® chest and limb modules were also added as a proof-of-concept demonstration. Table 2 shows demographic characteristics of validation study participants. All the assessed participants had an epilepsy related diagnosis, ranging from genetic syndromes to structural brain abnormalities such as cerebral injury from hypoxic ischemic encephalopathy. Recruitment included patients ages 0-18 who presented to the Lurie Epilepsy Monitoring Unit (EMU) as part of a clinically indicated 4-hour EEG study. There were no other exclusion criteria. Patients were screened by a pediatric epileptologist (J.N.W.) through review of the electronic medical record.”

2. We expanded upon the *Discussion* section to comment the increased sample size as follows:

“We present an innovative comprehensive wireless neurodevelopment monitoring system that adds neuronal activity measurement via EEG to previously validated chest and limb modules. Pilot studies at an academic quaternary pediatric care center demonstrate the practicality and feasibility of ANNE EEG to conduct electroencephalography studies with high levels of accuracy, validated via both quantitative and qualitative metrics, compared to the gold standard system. Furthermore, we also demonstrate the feasibility and scalability of the system for use in neonates in the LMIC setting. Previous work has already illustrated the performance and safety of the ANNE Chest and ANNE Limb sensors for cardiopulmonary monitoring in the LMIC setting [20,35,36].”

2. If not possible, then analyze other signals in addition to EEG that was presented in the current manuscript. Maybe ECG analysis including HRV could be included. Also, power spectrum analysis of EEG signals should be included to confirm the spikes. Other bands should also be investigated.

**Author response**: We appreciate the feedback and agree that the manuscript would be strengthened by additional data analysis. As such, we performed full power spectral analysis on each sleep EEG study that was included in the initial submission and subsequently extracted physiologically relevant power bands. Per the reviewer’s suggestion, we highlighted the effects of epileptiform spikes on the power spectra. We demonstrated a trend towards increasing delta power for EEG studies that included spikes, in concordance with prior literature.

We are unable to analyze other physiologic signals other than EEG for the study cohort due to limitations in our study design; however, we agree that this would be an important aspect of a multimodal system and intend to do this in future work, as mentioned in the final paragraph of the *Discussion* section.

**Manuscript modifications:**

1. We displayed results of the multi-center feasibility study in figure 4, adding the subsection to *Results* entitled “The EEG system captures key elements of neonatal sleep in HIC and LMIC settings”:

“Figure 4 displays typical graphoelements of neonatal sleep activity measured by ANNE EEG. Analysis of quiet and active stages of neonatal sleep has been shown to predict neurodevelopmental outcome [21]. Figure 4A displays features of quiet sleep in the HIC setting, and figure 4B displays separate features of active sleep in the LMIC setting.”

2. We added figure 7 and supplementary table 1. These display the results of power spectrum analysis and subsequent statistical analysis, respectively.

We expanded the *The EEG system demonstrates high levels of accuracy via qualitative and quantitative metrics in a pediatric cohort* subsection of the results section, consisting of the following paragraph:

“Further quantitative analysis (see: Methods for details of power spectrum analysis) was also performed for patients whose sleep activity was recorded via ANNE EEG. Figure 7 compares the distribution of EEG power (absolute broadband power and relative delta frequency) in sleep without epileptiform spikes versus sleep with epileptiform spikes. These findings are concordant with prior studies [25,26] that showed an increase in both these metrics for individuals with epilepsy.”

Respectively, we also added the following paragraph to the *Data Analysis* subsection of the **Materials and Methods** section:

“Relative power spectrum analysis was calculated for each channel in a subset of the validation study cohort. Sleep epochs were chosen to minimize the effects of movement artifact. The same representative 30 minute epochs of continuous sleep were used as in the alpha-delta ratio calculations. Power spectra for each of the 16 channels were divided into the following physiologically relevant frequencies; delta (0.5 to 4 Hz), theta (4 to 8 Hz), alpha (8 to 13 Hz), and beta (13 to 30 Hz). Each of these frequency bands were then averaged across all channels along with the total power for the epoch (Supplementary Table 1). Boxplots were then generated to compare relative delta ratio and total power between the subgroup with epileptiform discharges versus the subgroup without epileptiform discharges.”

3. We expanded upon the *Discussion* section to comment the increased sample size as follows:

“We present an innovative comprehensive wireless neurodevelopment monitoring system that adds neuronal activity measurement via EEG to previously validated chest and limb modules. Pilot studies at an academic quaternary pediatric care center demonstrate the practicality and feasibility of ANNE EEG to conduct electroencephalography studies with high levels of accuracy, validated via both quantitative and qualitative metrics, compared to the gold standard system. Furthermore, we also demonstrate the feasibility and scalability of the system for use in neonates in the LMIC setting”

4. We modified the following paragraph of the *Discussion* section to account for our study’s increased sample size and limitations with the additional statistical analysis:

“Our validation study has a few limitations that require further investigation prior to widespread deployment, particularly in medically underserved regions. While 30 subjects is adequate for validation of a novel EEG device validation [16] this is a relatively low number; we intend to increase this within our own institution. We also intend to expand upon the feasibility studies performed at both HIC and LMIC settings with future iterations of the system. Smaller form factors and electrode solutions that more specifically meet the needs of premature and ultra-low birth weight infants [36] would also be ideal for this vulnerable population.

While the power spectra trends displayed in Figure 7 were not statistically significant due to the small subgroup sample size, results are consistent with prior literature investigating spectral power in patients with epilepsy. We intend to investigate this trend further in future studies.”

3. Include the analysis to look at the relationship between age (years) with other physiological and neurological features.

**Author response**: We are unable to analyze other physiologic signals other than EEG for the study cohort due to limitations in our study design; however, we agree that this would be an important aspect of a multimodal system and intend to do this in future work.

**Reviewer 2:**

Wong et. al submitted a manuscript which introduces a novel wireless monitoring system that adds EEG measurements to an existing multimodal wearable sensor platform that has been FDA cleared to measure cardiac activity, blood oxygenation, motion, and skin temperature.    Their goal was to validate the novel system by recording high quality cerebral activity via EEG during simultaneous standard EEG recording.   The included 30 pediatric patients at a single academic quaternary pediatric care center as part of their pilot study.
The authors did a wonderful job addressing the need for such a novel system, postulating its use in low to middle income countries. 
The authors detailed the components of the experimental EEG platform, ANNE ECG, which added 16 channel cerebral activity monitoring to an existing USA FDA cleared ANNE wireless monitoring platform.  Based their results they were able to successfully validate their results against standard EEG recordings.   Furthermore, in assessing patient/parent satisfaction, there as an overwhelming positive review.
I commend the authors in their work which pushes to advance the field using current wireless technology.

Points of clarification:
1/ For the 13 patients with abnormal EEG findings, could you discuss in a bit more detail how the 13 abnormal ANNE EEG varied from the gold stand?  Figure 3 demonstrated almost no variation.  Were the findings as consistent with patients with diffuse slowing and epileptiform discharges as well?

**Author response**: Thank you for your thoughtful feedback. We added language to the Results section describing that there were occasional variations to the gold standard for some of the study cohort. This is also demonstrated by the close, but not perfect, correlation in the validation signal analysis seen in Figure 5.

**Manuscript modifications:**

1. We added clarifying text to the *The EEG system demonstrates high levels of accuracy via qualitative and quantitative metrics in a pediatric cohort* subsection of the Results section:

“On visual inspection, concurrent outputs between the two systems were similar for all patients, independent of their EEG findings. However, a few differences were noted. The ANNE EEG system’s outputs displayed a slightly sharper contour to their waveforms, likely owing to its higher sampling frequency (1000 Hz) vs that of the gold-standard system (512 Hz). In addition, at times there was some discordance in either phase or amplitude, likely due to the gold standard and ANNE EEG electrodes being separate and spatially separated by approximately 1cm. The electrode differences resulted in disparities likely due to single electrode artifact, variations in skin-electrode adhesion, or nonuniform effects of head movement artifact.”

2/ Practically speaking, if this system is meant market to LIMIC, what would be the assumed cost of such a system?   What is the project battery life?  Are any portion of the module reusable?
Minor revisions/recommendations:
Line 143.  The electronics “are” (switch “is” to “are”)

**Author response**: We wholeheartedly agree that in order for the system to be truly useful to the LMIC population, minimizing cost is crucial. We added language in the Results section on the current assumed cost and projected battery life. We also clarified which portions of the system are reusable.

**Manuscript modifications:**

1. We expanded content in the *Results* section, highlighting further technical details:

“Figure 1 displays key aspects of the system’s sensor design. A four-layer flexible printed circuit board is fabricated for the ANNE EEG sensor that consists of a stack-up of copper, polyimide, coverlay, and soldermask. The sensor consists of the following; a system-on-a-chip (SoC, ISP1807, Insight SIP); high-precision and low noise analog-front-ends (AFE) for a maximum of 16 channels; and a power management unit for wireless charging and supplying power to the on-board electronics with a rechargeable Li-polymer battery (230 mAh). The SoC controls the two AFE with on-board low-noise PGAs and sigma delta ADCs to collect clinical grade EEG data, where each channel samples at 1 kHz with 24-bit resolution and 0.28 µVrms input noise. The sensor board is enclosed with a ruggedized plastic housing made of Acrylonitrile butadiene styrene (ABS) with 16 channel connectors to standard EEG electrodes.”

2. We added the following text to the start of second paragraph of the Results section:

“Panel A of Figure 2 provides an exploded schematic view of the device to highlight the underlying technology. ANNE EEG has a battery life of 8 hours between charges and a projected cost of $1,000 USD to allow for cost-effective operation in LMIC environments. The device is fully reusable.”

3. We switched “is” to “are” in line 143.
