# Supplementary material for: A comprehensive wireless neurological and cardiopulmonary monitoring platform for pediatrics
Source: PLOS Digit Health. 2023 Jul 6;2(7):e0000291. doi: 10.1371/journal.pdig.0000291 (PMC10325120; doi:10.1371/journal.pdig.0000291)
Supplement: Attachment — Submitted filename: Rebuttal Xu PLOS Digital Health May2023.docx [file pdig.0000291.s003.docx]

**Rebuttal to Resubmission Responses from the Editors and Reviewers**

We would like to sincerely thank you for having taken time to review our resubmission and continued valuable feedback. We believe our responses here reflect a substantial revision of the manuscript making it suitable for publication in *PLOS Digital Health*.

**Editor Comments**

1. Figure 2 includes an image of an identifiable person. Please provide written confirmation or release forms, signed by the subject(s) (or their parent/legally authorized guardian), giving permission to be photographed and to have their images published under our CC-BY 4.0 license. Otherwise, we kindly request that you remove the photograph.

**Author response**: We obtained written consent for the subject’s parent giving permission to be photographed and published using the “Consent Form for Publication in a PLOS Journal” document supplied from PLOS.

**Manuscript modifications:** We attached the “Consent Form for Publication in a PLOS Journal” for Figure 2 in our resubmission.

2. The manuscript was largely improved by the authors, but a comment from a Reviewer, which I also assume as relevant, was disregarded, surely for a misunderstanding on the kind of analysis. Could the authors answer this comment in a revised version of the manuscript.

**Author response**: Thank you again for your continued detailed consideration of the manuscript. As a result of your and Reviewer #1’s feedback, we strengthened the data analysis further and added the resultant language and figure to the Results section. Reviewer #1 provided welcome clarity which allowed us to perform further data analysis as seen in Fig. 8.

**Manuscript modifications:**

1. We expanded content in the *Results* section to perform the additional analysis specifically requested by Reviewer #1 as follows:

“In addition to successful EEG signal validation, key neurodevelopmental metrics were also investigated. For the validation cohort without epileptiform activity in sleep, Figure 8 compares their age versus distribution of EEG band power in all physiologic frequencies (delta, theta, alpha, beta and gamma) via simple linear regression analysis. A strong negative correlation was demonstrated between age and delta power, and a strong positive correlation was likewise demonstrated between age and alpha power. These findings are concordant with what has previously been reported (ref) in neurodevelopmental studies.”

2. We added the following sentences to the last paragraph of the *Data Analysis* subsection in the **Materials and Methods** section:

“For the subgroup without epileptiform discharges, simple linear regression analysis was used to determine the correlation between validation subject age and power spectra for each band. The subgroup with epileptiform discharges was excluded from this specific analysis as the aim was to analyze age as a primary variable in neurodevelopment without confounding factors (such as epileptiform spikes) that would otherwise affect the EEG power spectra.”

3. We added Figure 8, demonstrating the correlation of EEG band power with ages of validation study participants.

3. Moreover, I could not see Fig. 7 in the pdf of the manuscript. In the Results section, I would remove the subheading, in particular the long ones (second and third).

**Author response**: We agree with these points.

**Manuscript modifications:** We ensured that Fig. 7 was visible in the manuscript submission PDF. We removed the second and third subheadings entitled “The EEG system captures key elements of neonatal sleep in HIC and LMIC settings” and “The EEG system demonstrates high levels of accuracy via qualitative and quantitative metrics in a pediatric cohort” in the Results section, combining them in to a single “Neurodevelopment system data analysis” subheading.

4. A comment on the figures in the manuscript with some quantitative analysis would also be interesting.

**Author response:** We agree with this point.

**Manuscript modifications:**

1. We moved the following sentences from the **Methods** section to the **Results** section to provide further context to the figures as well as their underlying quantitative analysis:

“Quantitative data analysis was performed for EEG studies recorded from the 30 subjects that comprised the validation study cohort. Similar analysis for the other 61 subjects in the feasibility arm could not be performed due to the lack of varied ages and control EEG data. For patients who fell asleep during EEG recording or had epileptiform discharges, 30 minute epochs were identified via manual inspection that had either continuous sleep or epileptiform discharges by a board-certified Child Neurologist (J.N.W.). From these, two quantitative EEG measures, alpha-delta ratio, and spike count were performed. Alpha-delta ratio is a validated marker of sleep [38,39]. Epileptiform spikes are a biomarker of seizures and epilepsy [40,41]. Automated analysis for both metrics were performed via a previously validated software package using MATLAB (Mathworks, California USA) software [42].

Relative power spectrum analysis was calculated for each channel in the validation study cohort. Sleep epochs were chosen to minimize the effects of movement artifact. The same representative 30 minute epochs of continuous sleep were used as in the alpha-delta ratio calculations. Power spectra for each of the 16 channels were divided into the following physiologically relevant frequencies; delta (0.5 to 4 Hz), theta (4 to 8 Hz), alpha (8 to 13 Hz), beta (13 to 30 Hz), and gamma (30Hz to 50Hz). Each of these frequency bands were then averaged across all channels along with the total power for the epoch (Table 1).”

2. We moved Supplementary Table 1 entitled “Relative power spectrum for each frequency band and absolute total power in validation study subjects in whom sleep was captured.” to Table 1 of the main body of the manuscript.
3. We added gamma frequency to the quantitative analysis as shown in Table 3.

**Reviewer 1:**

1. Thank you for responding to my comments and revising the manuscript. However my comment 3 is still not addressed. Please include a figure showing correlation of EEG band power (alpha, beta, theta and gamma) with ages (years) of the participants. Otherwise I would not be able to understand how neurodevelopment can be explained by your study design.

**Author response**: Thank you for your continued detailed feedback, in particular your clarification of the desired figure. As a result, we further strengthened the data analysis and added the resultant language and figure to the Results section.

**Manuscript modifications:**

1. We expanded content in the *Results* section to perform the additional analysis:

“In addition to successful EEG signal validation, key neurodevelopmental metrics were also investigated. For the validation cohort without epileptiform activity in sleep, Figure 8 compares their age versus distribution of EEG band power in all physiologic frequencies (delta, theta, alpha, beta and gamma) via simple linear regression analysis. A strong negative correlation was demonstrated between age and delta power, and a strong positive correlation was likewise demonstrated between age and alpha power. These findings are concordant with what has previously been reported (ref) in neurodevelopmental studies.”

2. We added the following sentences to the last paragraph of the *Data Analysis* subsection in the **Materials and Methods** section:

“For the subgroup without epileptiform discharges, simple linear regression analysis was used to determine the correlation between validation subject age and power spectra for each band. The subgroup with epileptiform discharges was excluded from this specific analysis as the aim was to analyze age as a primary variable in neurodevelopment without confounding factors (such as epileptiform spikes) that would otherwise affect the EEG power spectra.”

3. We added Figure 8, demonstrating the correlation of EEG band power with ages of validation study participants.
4. We added gamma frequency to the quantitative analysis as shown in Table 1.
2. Also I could not find analysis results of additional data in the figures.

**Author response**: Thank you for this feedback. Ideally, we would have liked to perform additional quantitative analysis for the additional 61 patients that were included in the resubmission. Unfortunately, due to the limitations of the feasibility study, specifically the lack of non-neonate ages for neurodevelopmental comparison and lack of control EEG data to definitively conclude sleep, this could not be accomplished. We agree that further analysis in a larger cohort is critical; as such, we intend to use the neurodevelopmental correlations demonstrated in the validation cohort as a basis for future work.

**Manuscript modifications:**

1. We added the following language to the **Results** section:

“Similar analysis for the other 61 subjects in the feasibility arm could not be performed due to the lack of varied ages and control EEG data.”
